# Supplementary material for: Can We Discover Truffle’s True Identity?
Source: Molecules. 2020 May 8;25(9):2217. doi: 10.3390/molecules25092217 (PMC7248893; doi:10.3390/molecules25092217)

Table S1. The summary on geo-environmental information of sampling locations and date of harvesting for *Tuber* species (*n* = 58) including latitude, longitude, altitude, climatic conditions, soil geology, geological age and host tree. The data for temperature and amount of precipitation were obtained by the Slovenian Environmental Agency of the Ministry of the Environment and Spatial Planning of the Republic of Slovenia. (n.d. – no data available)

**Species**	**Harvest time**	**Country**	**Location**	**Latitude**	**Longitude**	**Altitude** [m]	**Annual mean** T [°C]	**Annual mean precipitation** [mm]	**Soil geology**	**Geological age**	**Host tree**
TUBAES	14.11.2018	BIH	Šipovo (Location 1)	44.2837	17.0866	526	n.d.	n.d.	flysch; clastic-carbonate flysch	Jurassic-Cretaceous; late Cretaceous	*Corylus avellana, Carpinus betulus, Quercus robur*
TUBAES	14.11.2018	BIH	Šipovo (Location 2)	44.2837	17.0866	526	n.d.	n.d.	flysch; clastic-carbonate flysch	Jurassic-Cretaceous; late Cretaceous	*Corylus avellana, Carpinus betulus, Quercus robur*
TUBAES	07.11.2018	CRO	n.d.	n.d.	n.d.	n.d.	n.d.	n.d.	shallow-marine carbonate rocks	Cretaceous (Mesozoic)	n.d.
TUBAES	xx.02.2019	IT	Perugia	43.1105	12.3910	493	n.d.	n.d.	porous or fissured limestone; calcareous sandstones	Tertiary	n.d.
TUBAES	xx.02.2019	IT	Perugia	43.1105	12.3910	493	n.d.	n.d.	porous or fissured limestone; calcareous sandstones	Tertiary	n.d.
TUBAES	14.11.2018	MK	Bistra Mountain	41.6232	20.7340	2163	n.d.	n.d.	limestone	Paleozoic	*Fagus sylvatica*
TUBAES	10.11.2018	MK	Korab	41.8150	20.5676	2764	n.d.	n.d.	shale; limestone; gypsum rocks	Paleozoic; Permo Triassic	*Fagus sylvatica*
TUBAES	xx.10.2018	PL	n.d.	n.d.	n.d.	n.d.	n.d.	n.d.	rendzinas on marlstone, limestone, gypsum	Tertiary-Quaternary (Cenozoic)	*Carpinus betulus*
TUBAES	xx.10.2018	PL	n.d.	n.d.	n.d.	n.d.	n.d.	n.d.	rendzinas on marlstone, limestone, gypsum	Tertiary-Quaternary (Cenozoic)	*Corylus avellana*
TUBAES	xx.10.2018	PL	n.d.	n.d.	n.d.	n.d.	n.d.	n.d.	rendzinas on marlstone, limestone, gypsum	Tertiary-Quaternary (Cenozoic)	*Quercus robur*
TUBAES	20.09.2018	SLO	Bloke	45.7731	14.5094	729	9.1	1403	shallow-marine predominantly carbonate rocks	Jurassic-Triassic (Mesozoic)	*Corylus avellana, Betula pendula*
TUBAES	20.09.2018	SLO	Bloke	45.7731	14.5094	729	9.1	1403	shallow-marine predominantly carbonate rocks	Jurassic-Triassic (Mesozoic)	*Corylus avellana, Betula pendula*
TUBAES	26.09.2018	SLO	Bloke	45.7731	14.5094	729	9.1	1403	shallow-marine predominantly carbonate rocks	Jurassic-Triassic (Mesozoic)	*Corylus avellana, Betula pendula*
TUBAES	26.09.2018	SLO	Bloke	45.7731	14.5094	729	9.1	1403	shallow-marine predominantly carbonate rocks	Jurassic-Triassic (Mesozoic)	*Corylus avellana, Betula pendula*
TUBAES	03.11.2018	SLO	Meja	46.1913	14.3765	265	10.7	1410	terrestrial deposits	Quaternary (Cenozoic)	*Quercus robur, Carpinus betulus*
TUBAES	13.10.2018	SLO	Pluska	45.9120	14.9603	320	11.8	1106	rendzinas and brown soils on limestone and dolomite	Jurassic (Mesozoic); Jurassic-Triassic (Mesozoic)	*Carpinus betulus, Fagus sylvatica, Picea abies*
TUBAES	30.08.2018	SLO	Rajndol	45.5678	14.9475	510	10.0	1485	shallow-marine predominantly carbonate rocks	Jurassic (Mesozoic); Jurassic-Triassic (Mesozoic)	*Corylus avellana, Carpinus betulus*
TUBAES	30.08.2018	SLO	Rajndol	45.5678	14.9475	510	10.0	1485	shallow-marine predominantly carbonate rocks	Jurassic (Mesozoic); Jurassic-Triassic (Mesozoic)	*Carpinus betulus*
TUBAES	03.11.2018	SLO	Sežana	45.7034	13.8706	360	10.5	1698	eutric brown soils, calcaric on flysch	Cretaceous (Mesozoic)	*Quercus pubescens, Corylus avellana*
TUBAES	03.11.2018	SLO	Sežana	45.7034	13.8706	360	10.5	1698	eutric brown soils, calcaric on flysch	Cretaceous (Mesozoic)	*Tilia platyphilla, Ostrya carpinifolia*
TUBAES	16.10.2018	SLO	Sežana	45.7034	13.8706	360	10.5	1698	eutric brown soils, calcaric on flysch	Cretaceous (Mesozoic)	*Quercus pubescens, Corylus avellana*
TUBAES	27.08.2018	SLO	Sežana	45.7034	13.8706	360	10.5	1698	eutric brown soils, calcaric on flysch	Cretaceous (Mesozoic)	*Pinus nigra, Quercus pubescens*
TUBAES	27.08.2018	SLO	Sežana	45.7034	13.8706	360	10.5	1698	eutric brown soils, calcaric on flysch	Cretaceous (Mesozoic)	*Pinus nigra, Quercus pubescens*
TUBAES	28.08.2018	SLO	Sežana	45.7034	13.8706	360	10.5	1698	eutric brown soils, calcaric on flysch	Cretaceous (Mesozoic)	*Quercus pubescens, Corylus avellana*
TUBAES	06.10.2018	SLO	Snežna jama	45.6541	15.0270	875	8.3	1330	rendzic leptosol on carbonate bedrock	Cretaceous (Mesozoic)	*Fagus sylvatica, Abies alba*
TUBAES	03.11.2018	SLO	Spodnje Blato	45.9554	14.6817	338	11.3	1332	shallow-marine predominantly carbonate rocks	Quaternary (Cenozoic); Jurassic-Triassic (Mesozoic)	*Carpinus betulus, Fagus sylvatica, Quercus robur*
TUBAES	13.10.2018	SLO	Spodnje Blato	45.9554	14.6817	338	11.3	1332	brown soils on limestone and dolomite	Quaternary (Cenozoic); Jurassic-Triassic (Mesozoic)	*Quercus robur*
TUBAES	13.10.2018	SLO	Spodnje Blato	45.9554	14.6817	338	11.3	1332	brown soils on limestone and dolomite	Quaternary (Cenozoic); Jurassic-Triassic (Mesozoic)	*Carpinus betulus, Fagus sylvatica, Quercus robur*
TUBAES	13.10.2018	SLO	Stari Log	45.7254	14.9223	400	10.0	1485	brown soils on limestone and dolomite	Cretaceous (Mesozoic)	*Corylus avellana, Carpinus betulus*
TUBAES	06.10.2018	SLO	Žlebič	45.7660	14.6931	512	7.9	1380	shallow-marine carbonate and clastic rocks	Carboniferous (Paleozoic); Triassic (Mesozoic)	*Corylus avellana, Carpinus betulus*
TUBAES	09.09.2018	SLO	Žlebič	45.7660	14.6931	512	7.9	1380	shallow-marine carbonate and clastic rocks	Carboniferous (Paleozoic); Triassic (Mesozoic)	*Quercus robur, Carpinus betulus*
TUBAES	09.09.2018	SLO	Žlebič	45.7660	14.6931	512	7.9	1380	shallow-marine carbonate and clastic rocks	Carboniferous (Paleozoic); Triassic (Mesozoic)	*Quercus robur, Carpinus betulus*
TUBAES	19.08.2018	SLO	Žlebič	45.7660	14.6931	512	7.9	1380	shallow-marine carbonate and clastic rocks	Carboniferous (Paleozoic); Triassic (Mesozoic)	*Quercus robur, Carpinus betulus*
TUBAES	19.08.2018	SLO	Žlebič	45.7660	14.6931	512	7.9	1380	shallow-marine carbonate and clastic rocks	Carboniferous (Paleozoic); Triassic (Mesozoic)	*Quercus robur, Carpinus betulus*
TUBAES	19.08.2018	SLO	Žlebič	45.7660	14.6931	512	7.9	1380	shallow-marine carbonate and clastic rocks	Carboniferous (Paleozoic); Triassic (Mesozoic)	*Quercus robur, Carpinus betulus*
TUBAES	19.08.2018	SLO	Žlebič	45.7660	14.6931	512	7.9	1380	shallow-marine carbonate and clastic rocks	Carboniferous (Paleozoic); Triassic (Mesozoic)	*Quercus robur, Carpinus betulus*
TUBAES	30.08.2018	SLO	Žlebič	45.7660	14.6931	512	7.9	1380	shallow-marine carbonate and clastic rocks	Carboniferous (Paleozoic); Triassic (Mesozoic)	*Quercus robur, Carpinus betulus*
TUBAES	30.08.2018	SLO	Žlebič	45.7660	14.6931	512	7.9	1380	shallow-marine carbonate and clastic rocks	Carboniferous (Paleozoic); Triassic (Mesozoic)	*Quercus robur, Carpinus betulus*
TUBAES	30.08.2018	SLO	Žlebič	45.7660	14.6931	512	7.9	1380	shallow-marine carbonate and clastic rocks	Carboniferous (Paleozoic); Triassic (Mesozoic)	*Quercus robur, Carpinus betulus*
TUBBRU	19.11.2018	CRO	Krčenik - Donji Miholjac	45.7494	17.9695	97	n.d.	n.d.	clastic deposits	Holocene	*Carpinus betulus, Quercus robur, Corylus avellana*
TUBBRU	27.10.2018	SLO	Marija Snežna	45.7661	14.1932	467	10.8	1426	brown soils/rendzina on limestone and dolomite	Jurassic-Triassic (Mesozoic)	*Tilia platyphilla*
TUBIND	10.01.2019	CN	n.d.	n.d.	n.d.	n.d.	n.d.	n.d.	igneous crystal rocks or metamoprhic rocks/cambisols	Quaternary (Cenozoic)	n.d.
TUBIND	10.01.2019	CN	n.d.	n.d.	n.d.	n.d.	n.d.	n.d.	igneous crystal rocks or metamoprhic rocks/cambisols	Quaternary (Cenozoic)	n.d.
TUBIND	10.01.2019	CN	n.d.	n.d.	n.d.	n.d.	n.d.	n.d.	igneous crystal rocks or metamoprhic rocks/cambisols	Quaternary (Cenozoic)	n.d.
TUBMAC	19.11.2018	CRO	Krčenik - Donji Miholjac	45.7494	17.9695	97	n.d.	n.d.	clastic deposits	Holocene	*Carpinus betulus, Quercus robur, Corylus avellana*
TUBMAG	01.01.2019	IT	Perugia	43.1105	12.3910	493	n.d.	n.d.	shallow-marine predominantly carbonate rocks	Jurassic-Triassic (Mesozoic)	n.d.
TUBMAG	15.12.2018	SLO	Glem	45.4887	13.7829	302	14.8	873	flysch and other deep-marine rocks	Paleogene (Cenozoic)	n.d.
TUBMAG	15.12.2018	SLO	Lukini	45.4722	13.8961	321	14.8	873	flysch and other deep-marine rocks	Paleogene (Cenozoic)	n.d.
TUBMAG	15.12.2018	SLO	Vanganel	45.5171	13.7780	34	14.8	873	flysch and other deep-marine rocks	Paleogene (Cenozoic)	*Populus tremula, Quercus* spp.
TUBMEL	18.01.2019	ES	Cantavieja	40.5256	-0.4059	1290	n.d.	n.d.	leptic cambisol; limestone, calcareous soils on shales	Cretaceous (Mesozoic)	n.d.
TUBMEL	15.01.2019	ES	n.d.	n.d.	n.d.	n.d.	n.d.	n.d.	n.d.	n.d.	n.d.
TUBMEL	20.01.2019	ES	n.d.	n.d.	n.d.	n.d.	n.d.	n.d.	n.d.	n.d.	n.d.
TUBMEL	xx.02.2019	IT	Perugia	43.1105	12.3910	493	n.d.	n.d.	porous or fissured limestone; calcareous sandstones	Tertiary	n.d.
TUBMEL	xx.02.2019	IT	Perugia	43.1105	12.3910	493	n.d.	n.d.	porous or fissured limestone; calcareous sandstones	Tertiary	n.d.
TUBMES	xx.02.2019	IT	Perugia	43.1105	12.3910	493	n.d.	n.d.	porous or fissured limestone; calcareous sandstones	Tertiary	n.d.
TUBMES	xx.12.2018	MK	n.d.	n.d.	n.d.	n.d.	n.d.	n.d.	n.d.	n.d.	n.d.
TUBMES	09.11.2018	MK	Šar Mountains	42.0844	20.8332	2748	n.d.	n.d.	limestone rocks; stony ground	n.d.	*Fagus sylvatica*
TUBMES	09.11.2018	MK	Šar Mountains	42.0844	20.8332	2748	n.d.	n.d.	limestone rocks; stony ground	n.d.	*Fagus sylvatica*

Table S2. The content of elements (mg/kg) in the peridial layer of fruiting bodies of *Tuber* species (n = 58). (n.d. – no data available)

Species	Country	Location	Al	As	Ba	Ca	Cd	Co	Cr	Cs	Cu	Fe	Hg	K	Mg	Mn	Na	Ni	P	Pb	Rb	S	Sr	V	Zn
TUBAES	BIH	Šipovo (Location 1)	1158	0.43	11.1	3048	10.6	469	4.46	0.17	116	632	0.11	20078	1035	26.5	125	3.91	5703	0.55	6.79	2087	7.19	2.52	174
TUBAES	BIH	Šipovo (Location 2)	157	0.29	2.44	2843	1.80	115	0.85	0.08	85.2	130	0.10	17492	714	8.33	95.7	0.91	3867	0.13	2.77	2067	5.13	0.34	123
TUBAES	CRO	n.d.	264	0.13	9.24	2210	9.41	77.1	2.64	0.03	32.1	159	0.05	24739	1515	30.8	78.3	0.57	4051	0.54	16.7	2389	2.54	0.61	269
TUBAES	IT	Perugia	362	0.09	6.81	2740	6.00	120	1.08	0.04	79.4	258	0.05	16820	930	10.8	60.8	0.54	6801	0.13	1.24	2552	8.21	0.75	132
TUBAES	IT	Perugia	170	0.05	5.29	3406	3.54	70	1.24	0.03	39.4	118	0.12	18239	855	8.31	80.9	0.34	3747	0.20	8.68	1739	4.35	0.41	183
TUBAES	MK	Bistra Mountain	181	0.08	3.61	1548	2.62	61.0	0.65	0.02	27.7	130	0.07	24203	761	14.8	108	0.25	6915	0.72	5.84	1560	1.82	0.38	137
TUBAES	MK	Korab	1128	0.72	14.9	2591	5.61	342	2.52	0.12	69.9	546	0.06	22204	1230	31.9	76.4	1.34	5674	0.69	2.66	1872	3.54	2.09	114
TUBAES	PL	n.d.	273	0.13	7.67	4804	5.83	133	0.57	0.03	22.0	223	0.04	16792	619	13.9	65.9	0.37	4596	0.45	1.93	3026	23.8	0.85	120
TUBAES	PL	n.d.	88	0.08	6.27	3424	3.27	54.2	0.44	0.01	25.9	60.0	0.03	18923	521	5.42	43.1	0.57	3610	0.25	3.62	3310	18.7	0.31	118
TUBAES	PL	n.d.	808	0.23	12.4	3443	4.28	201	1.40	0.08	39.7	444	0.10	22741	858	16.0	75.6	1.14	5610	0.92	4.77	3677	18.8	1.72	184
TUBAES	SLO	Bloke	552	0.25	4.65	2536	7.95	153	1.61	0.05	31.0	411	0.08	23547	1652	16.0	139	0.94	4469	0.80	14.0	1808	2.20	2.20	127
TUBAES	SLO	Bloke	291	0.09	9.23	2503	12.4	116	0.95	0.03	48.3	198	0.20	22426	1131	15.6	91.6	0.44	4571	0.22	4.52	1658	2.71	0.54	180
TUBAES	SLO	Bloke	1640	0.57	7.45	2522	9.70	413	3.40	0.16	23.9	1215	0.03	17641	1608	29.6	98.8	2.03	2306	0.88	13.4	1778	2.67	4.95	55.0
TUBAES	SLO	Bloke	1081	0.39	6.67	2685	11.3	291	2.68	0.11	30.1	656	0.06	21775	1911	30.0	108	1.52	3879	0.69	28.2	2204	2.75	3.70	78.1
TUBAES	SLO	Meja	578	0.21	7.88	2576	6.49	114	1.51	0.07	48.6	429	0.13	16305	775	17.9	75.2	0.59	2067	0.78	5.79	3350	3.13	1.08	182
TUBAES	SLO	Pluska	332	0.16	4.52	2290	15.4	171	1.69	0.05	43.0	214	0.06	18935	1307	12.8	90.8	0.86	4903	0.30	4.54	1572	2.28	0.91	178
TUBAES	SLO	Rajndol	142	0.06	4.67	1702	5.04	63.7	1.31	0.02	45.8	100	0.05	24811	1300	10.6	210	0.41	3922	0.16	8.21	1528	1.75	0.39	136
TUBAES	SLO	Rajndol	179	0.07	6.97	1979	6.89	65.9	1.49	0.03	47.2	119	0.05	26212	1426	10.7	211	0.53	3804	0.14	12.9	1625	1.75	0.43	115
TUBAES	SLO	Sežana	492	0.28	4.46	3612	3.02	220	3.49	0.11	35.0	344	0.05	17242	685	18.4	52.0	1.28	3072	0.41	6.05	1537	2.76	2.13	105
TUBAES	SLO	Sežana	168	0.10	3.46	2689	3.77	155	4.07	0.03	27.0	121	0.07	15464	798	7.40	227	0.63	3192	0.24	9.03	2818	2.80	0.56	161
TUBAES	SLO	Sežana	1103	0.61	4.93	3757	4.00	322	5.13	0.23	38.8	719	0.06	21423	961	26.7	92.6	2.34	3691	0.66	6.60	1507	3.35	3.34	119
TUBAES	SLO	Sežana	1711	0.52	6.26	3132	3.72	482	5.09	0.21	37.4	498	0.01	18931	1081	36.3	153	2.88	4465	1.28	27.6	1628	3.72	3.56	142
TUBAES	SLO	Sežana	1015	0.27	5.32	2634	3.40	268	2.66	0.14	36.7	665	0.08	20531	827	21.9	83.5	1.57	6711	0.93	21.3	2183	3.43	2.03	88.7
TUBAES	SLO	Sežana	3710	1.77	15.2	5135	5.18	1014	8.79	0.71	61.0	1215	0.12	22455	2718	87.3	139	6.90	5657	2.33	13.9	1951	7.32	9.66	163
TUBAES	SLO	Snežna jama	563	0.21	6.38	3123	13.0	196	2.51	0.06	32.7	397	0.04	18102	1053	19.6	135	0.81	4468	0.57	7.29	2101	2.26	1.08	147
TUBAES	SLO	Spodnje Blato	467	0.17	6.68	3056	1.78	276	2.78	0.06	36.3	304	0.09	18666	1272	22.1	109	0.35	2642	0.29	12.8	1430	2.21	1.02	135
TUBAES	SLO	Spodnje Blato	276	0.10	6.42	3164	2.05	113	1.40	0.04	39.4	154	0.08	24370	1490	15.5	102	2.14	3931	0.16	12.8	1739	2.66	0.52	135
TUBAES	SLO	Spodnje Blato	296	0.10	6.51	2905	2.28	198	2.30	0.04	48.7	180	0.04	22997	1454	18.0	43.3	0.27	2530	0.21	14.3	1395	2.44	0.55	128
TUBAES	SLO	Stari Log	710	0.22	7.81	3447	5.29	205	1.83	0.07	37.7	340	0.13	21547	886	19.4	122	0.89	3972	0.46	9.15	1459	4.18	1.59	109
TUBAES	SLO	Žlebič	256	0.09	9.37	3577	6.51	105	2.48	0.04	58.4	170	0.09	22810	1201	15.8	84.6	0.59	3716	0.32	6.16	1850	3.33	0.54	135
TUBAES	SLO	Žlebič	291	0.08	6.51	3030	7.84	92.3	2.44	0.04	58.8	192	0.05	20263	940	11.4	103	0.51	4160	0.33	9.24	1726	2.60	0.57	121
TUBAES	SLO	Žlebič	216	0.07	4.11	1470	4.56	69.5	2.62	0.02	39.5	166	0.07	20928	846	8.73	58.3	0.27	3197	0.17	5.54	2416	1.45	0.36	111
TUBAES	SLO	Žlebič	215	0.07	8.33	3054	7.41	57.2	3.69	0.03	71.8	120	0.10	21961	1537	14.8	100	0.36	4847	0.18	6.91	2090	2.87	0.48	167
TUBAES	SLO	Žlebič	81.1	0.03	4.18	1850	8.76	47.4	1.71	0.01	55.2	55.0	0.06	25471	857	8.65	50.6	0.35	4750	0.13	3.68	2033	1.28	0.21	139
TUBAES	SLO	Žlebič	29.0	0.03	1.20	668	9.02	36.1	1.03	0.01	35.4	27.0	0.06	32571	1100	5.72	138	1.00	8285	0.07	5.26	2357	0.58	0.07	133
TUBAES	SLO	Žlebič	984	0.25	6.58	2658	8.57	313	3.22	0.10	65.4	559	0.07	20738	1013	16.8	149	1.14	3729	0.88	14.0	1757	2.73	1.54	86.1
TUBAES	SLO	Žlebič	116	0.05	5.69	2650	7.69	29.9	1.29	0.02	50.1	63.0	0.05	27086	1139	9.23	146	0.25	4849	0.08	5.32	1735	2.28	0.24	135
TUBAES	SLO	Žlebič	180	0.06	6.26	2870	4.86	41.7	3.25	0.02	48.2	117	0.04	20410	981	6.92	88.3	0.33	2946	0.13	3.88	1786	2.03	0.43	106
TUBAES	SLO	Žlebič	379	0.13	9.38	3069	2.76	76.1	1.67	0.05	46.6	210	0.12	25332	1159	8.04	123	0.23	3142	0.51	3.67	1251	3.14	0.65	139
TUBBRU	CRO	Krčenik - Donji Miholjac	1418	0.23	17.8	4325	6.20	291	2.13	0.13	31.9	831	0.05	20956	2093	16.0	617	1.35	6790	0.70	10.0	1376	15.9	2.16	225
TUBBRU	SLO	Marija Snežna	1335	0.32	5.09	980	12.5	356	4.50	0.14	145	462	0.02	31458	994	33.0	145	1.76	10181	0.65	25.7	4638	2.98	3.02	143
TUBIND	CN	n.d.	104	0.04	2.63	1285	0.96	154	1.50	0.02	19.1	84.4	0.08	17051	731	12.0	58.4	0.51	6027	0.33	1.16	3649	4.92	0.27	60.7
TUBIND	CN	n.d.	52.6	0.06	2.08	930	2.51	73.4	0.62	0.01	46.4	43.2	0.05	30429	646	7.61	73.5	0.27	10149	0.08	1.65	2114	3.77	0.13	105
TUBIND	CN	n.d.	22.4	0.04	1.26	747	0.42	77.9	0.51	0.01	17.1	24.5	0.06	23548	740	6.8	41.1	0.31	6183	0.04	1.70	2701	2.42	0.11	61.6
TUBMAC	CRO	Krčenik - Donji Miholjac	1961	0.28	21.7	3868	7.90	360	2.62	0.19	29.8	589	0.03	23702	2268	20.8	354	2.18	6965	1.01	16.7	1230	12.0	3.37	298
TUBMAG	IT	Perugia	715	0.16	3.91	1220	0.77	330	1.82	0.08	72.7	387	0.04	34061	1087	15.1	213	1.36	10252	0.25	6.98	2354	4.95	1.48	345
TUBMAG	SLO	Glem	1038	0.16	5.97	2551	2.97	318	3.10	0.12	25.5	228	0.04	28339	813	16.4	138	2.75	7091	0.44	5.48	2749	5.36	2.15	275
TUBMAG	SLO	Lukini	3238	0.21	16.9	1238	2.78	958	8.41	0.34	55.2	1847	0.05	28203	1167	23.9	501	6.79	7860	1.08	14.5	1897	10.7	6.35	373
TUBMAG	SLO	Vanganel	1557	0.26	6.09	1366	2.15	543	5.22	0.16	94.2	352	0.04	35844	853	24.6	182	3.93	7632	0.57	12.8	5808	3.45	3.21	390
TUBMEL	ES	Cantavieja	3595	1.94	13.8	5418	2.82	946	1.06	0.41	40.4	2162	0.05	17820	849	56.3	111	3.15	7003	1.81	9.18	2552	7.34	7.26	83.1
TUBMEL	ES	n.d.	260	0.14	1.69	3037	0.49	143	0.84	0.04	32.9	204	0.03	23097	645	10.1	97.1	0.44	5730	0.27	1.81	4122	4.71	0.57	117
TUBMEL	ES	n.d.	113	0.03	1.08	3096	0.53	39.0	1.45	0.05	32.6	84.2	0.05	18194	515	5.36	27.0	0.15	5561	0.08	8.49	4048	0.81	0.25	93.5
TUBMEL	IT	Perugia	382	0.08	3.08	2527	3.90	369	1.52	0.04	270	222	0.08	20447	464	15.5	56.6	0.58	5870	0.25	2.15	4132	2.39	0.80	109
TUBMEL	IT	Perugia	178	0.04	2.69	3366	4.03	134	1.06	0.02	234	116	0.03	19418	360	9.39	68.5	0.39	6456	0.12	3.13	6630	2.36	0.38	98.9
TUBMES	IT	Perugia	148	0.04	3.56	2342	5.55	99.4	0.69	0.02	43.3	118	0.14	17194	1058	9.94	168	0.39	6101	0.35	4.88	1752	3.46	0.35	139
TUBMES	MK	n.d.	1559	0.49	13.2	3681	1.36	413	2.80	0.17	48.0	1104	0.12	20240	947	43.0	180	1.46	4210	1.7	15.3	1872	4.66	2.69	100
TUBMES	MK	Šar Mountains	192	0.14	4.87	1467	6.54	68.3	0.98	0.02	63.5	137	0.06	23499	1503	14.7	245	0.53	7330	0.59	15.6	2353	1.92	0.40	196
TUBMES	MK	Šar Mountains	143	0.11	4.03	1380	8.50	83.2	1.36	0.02	52.8	107	0.06	25824	1080	13.3	184	0.76	6739	0.22	8.07	1757	1.59	0.23	189

Table S3. Natural isotopic abundances of light elements (per mil, ‰) in *peridium* of the fruiting bodies of *Tuber* species (*n* = 58). (n.d. – no data available)

Species	Country	Location	δ^2H	$\delta^{13}C$	$\delta^{15}N$	$\delta^{18}O$	$\delta^{34}S$
TUBAES	BIH	Šipovo (Location 1)	n.d.	-26.6	11.3	n.d.	5.6
TUBAES	BIH	Šipovo (Location 2)	-24.3	-27.3	8.2	18.9	6.3
TUBAES	CRO	n.d.	-14.1	-27.4	5.7	19.5	7.5
TUBAES	IT	Perugia	-3.4	-27.9	7.9	20.3	0.2
TUBAES	IT	Perugia	-20.3	-25.1	4.4	19.2	10.8
TUBAES	MK	Bistra Mountain	7.8	-26.4	4.2	21.4	7.1
TUBAES	MK	Korab	-25.0	-25.6	11.0	18.5	7.4
TUBAES	PL	n.d.	-12.9	-26.3	3.0	19.4	4.9
TUBAES	PL	n.d.	-3.5	-27.1	1.8	19.6	5.2
TUBAES	PL	n.d.	-21.8	-25.4	4.7	18.6	5.1
TUBAES	SLO	Bloke	-28.9	-28.5	5.8	17.9	7.2
TUBAES	SLO	Bloke	-22.1	-26.2	7.4	18.5	6.2
TUBAES	SLO	Bloke	-17.0	-27.5	6.8	18.4	7.6
TUBAES	SLO	Bloke	-17.5	-27.2	6.2	19.9	8.1
TUBAES	SLO	Meja	-8.1	-26.5	3.9	19.8	8.7
TUBAES	SLO	Pluska	0.8	-26.6	4.2	19.9	4.9
TUBAES	SLO	Rajndol	-15.5	-25.7	5.9	19.6	6.2
TUBAES	SLO	Rajndol	-15.6	-25.5	8.3	18.7	6.5
TUBAES	SLO	Sežana	-6.3	-25.8	5.4	20.6	7.3
TUBAES	SLO	Sežana	-2.7	-25.2	6.2	20.8	8.7
TUBAES	SLO	Sežana	-4.8	-26.4	5.3	20.5	6.2
TUBAES	SLO	Sežana	2.2	-26.8	6.9	20.8	7.0
TUBAES	SLO	Sežana	14.8	-25.9	5.1	21.7	6.8
TUBAES	SLO	Sežana	-7.5	-26.3	6.8	20.2	6.9
TUBAES	SLO	Snežna jama	-18.8	-26.6	6.4	18.8	6.8
TUBAES	SLO	Spodnje Blato	-11.3	-27.5	7.7	19.3	5.9
TUBAES	SLO	Spodnje Blato	-22.3	-27.2	6.9	18.1	5.8
TUBAES	SLO	Spodnje Blato	-21.6	-27.3	7.3	18.7	5.6
TUBAES	SLO	Stari Log	-12.4	-25.8	7.0	19.0	6.5
TUBAES	SLO	Žlebič	-22.8	-26.3	6.7	18.3	5.6
TUBAES	SLO	Žlebič	-8.0	-25.5	5.1	19.4	5.4
TUBAES	SLO	Žlebič	-16.4	-26.3	7.3	19.7	6.2
TUBAES	SLO	Žlebič	-17.0	-26.3	7.3	19.6	7.9
TUBAES	SLO	Žlebič	-17.5	-26.0	6.6	17.4	6.3
TUBAES	SLO	Žlebič	-15.3	-26.0	6.6	19.3	6.5
TUBAES	SLO	Žlebič	-29.3	-26.3	7.1	18.0	6.9
TUBAES	SLO	Žlebič	-24.9	-25.9	8.2	18.1	6.9
TUBAES	SLO	Žlebič	-26.2	-26.6	7.4	18.9	7.0
TUBAES	SLO	Žlebič	-18.3	-26.9	4.5	18.7	6.7
TUBBRU	CRO	Krčenik - Donji Miholjac	-13.1	-26.3	8.2	18.9	-0.9
TUBBRU	SLO	Marija Snežna	2.9	-24.5	10.5	21.1	8.8
TUBIND	CN	n.d.	-56.0	-27.9	5.5	15.8	6.2
TUBIND	CN	n.d.	-36.9	-26.0	5.7	17.2	6.2
TUBIND	CN	n.d.	-49.3	-26.0	8.2	16.5	6.6
TUBMAC	CRO	Krčenik - Donji Miholjac	-14.8	-26.1	7.9	19.4	-0.7
TUBMAG	IT	Perugia	-15.5	-27.5	13.7	20.0	-15.4
TUBMAG	SLO	Glem	-19.1	-26.2	17.9	19.6	4.4
TUBMAG	SLO	Lukini	-5.4	-28.0	19.6	18.5	-3.2
TUBMAG	SLO	Vanganel	-4.0	-27.0	14.2	19.6	7.4
TUBMEL	ES	Cantavieja	-16.4	-28.2	7.4	21.4	7.8
TUBMEL	ES	n.d.	-16.0	-26.3	7.9	20.1	3.1
TUBMEL	ES	n.d.	-13.8	-25.1	9.6	20.0	11.2
TUBMEL	IT	Perugia	1.9	-25.2	8.7	19.6	11.2
TUBMEL	IT	Perugia	-2.5	-23.8	7.5	22.5	11.0
TUBMES	IT	Perugia	-14.6	-25.8	3.9	18.8	11.0
TUBMES	MK	n.d.	-19.0	-25.7	4.7	19.5	8.4
TUBMES	MK	Šar Mountains	-14.9	-26.1	4.6	19.8	7.5
TUBMES	MK	Šar Mountains	-6.3	-26.3	5.6	21.2	7.4

Table 4S. Protocol for effective Sr-matrix separation from the truffle samples.

Cycle	Volume of reagent	Description
Washing the resin	3 x 1 ml MQ	regenerating the column
	1 ml 6 M HCl	
	10 x 1 ml MQ	
Resin activation	3 x 1 ml 8 M HNO_3	impurities removal
	10 x 1 ml MQ	method blank
Sample loading	1 ml sample in 8 M HNO_3	
Rb elution	6 x 1 ml 8 M HNO_3	Rb removal
Sr elution	10 x 1 ml MQ	collection of Sr fractions

Figure S1. Basic scheme for the quantitative-XRF analysis.

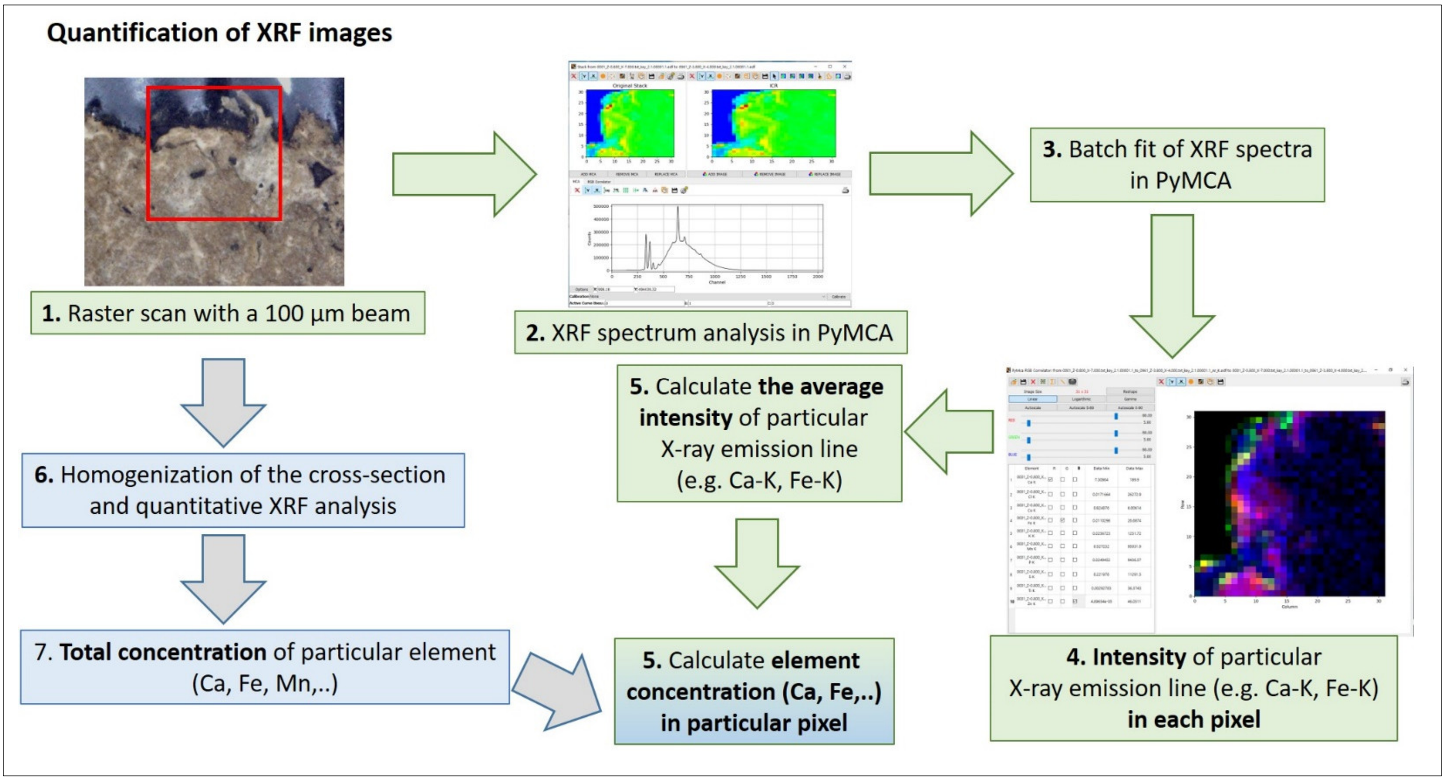

Supplement: Supplementary file 1 [file molecules-25-02217-s001.pdf]
